# Research on Evolution of Relevant Defects in Heavily Mg-Doped GaN by H Ion Implantation Followed by Thermal Annealing

**DOI:** 10.3390/ma17112518

**Published:** 2024-05-23

**Authors:** Zonglin Jiang, Dan Yan, Ning Zhang, Junxi Wang, Xuecheng Wei

**Affiliations:** 1 Research and Development Center for Wide Bandgap Semiconductors, Institute of Semiconductors, Chinese Academy of Sciences, Beijing 100083, China; jiangzl@semi.ac.cn (Z.J.); yandan@semi.ac.cn (D.Y.); zhangn@semi.ac.cn (N.Z.); jxwang@semi.ac.cn (J.W.); 2 College of Materials Science and Opto–Electronic Technology, University of Chinese Academy of Sciences, Beijing 100049, China

**Keywords:** GaN, heavily Mg-doped, H-related defect, nitrogen vacancy, ion implantation, thermal annealing

## Abstract

This study focuses on the heavily Mg-doped GaN in which the passivation effect of hydrogen and the compensation effect of nitrogen vacancies (V_N_) impede its further development. To investigate those two factors, H ion implantation followed by thermal annealing was performed on the material. The evolution of relevant defects (H and V_N_) was revealed, and their distinct behaviors during thermal annealing were compared between different atmospheres (N_2_/NH_3_). The concentration of H and its associated yellow luminescence (YL) band intensity decrease as the thermal annealing temperature rises, regardless of the atmosphere being N_2_ or NH_3_. However, during thermal annealing in NH_3_, the decrease in H concentration is notably faster compared to N_2_. Furthermore, a distinct trend is observed in the behavior of the blue luminescence (BL) band under N_2_ and NH_3_. Through a comprehensive analysis of surface properties, we deduce that the decomposition of NH_3_ during thermal annealing not only promotes the out-diffusion of H ions from the material, but also facilitates the repair of V_N_ on the surface of heavily Mg-doped GaN. This research could provide crucial insights into the post-growth process of heavily Mg-doped GaN.

## 1. Introduction

Gallium nitride (GaN), a pivotal wide-bandgap semiconductor, holds significant prominence in the field of optoelectronic and electronic devices due to its exceptional properties, including as a visible and ultraviolet light-emitting diode (LED), laser (LD), and high electron mobility transistor (HEMT) [1]. The wide applications of GaN are closely tied to the successful production of p-type GaN [2]. The p-type GaN serves as a hole injection layer to provide holes for the active region of LED and LD [3], while also serving as a gate electrode in HEMT to make it an enhancement-mode device [4].

At present, magnesium (Mg) stands as the primary acceptor dopant employed in p-type GaN. During the epitaxial growth process of metal–organic chemical vapor deposition (MOCVD), it is inevitable that a large number of hydrogen (H) atoms are introduced into Mg-doped GaN via the metal–organic precursor, NH_3_ and H_2_, which serves as carrier gas. Those H atoms tend to interact with the Mg acceptor to form the Mg-H complex, impeding the activation of the Mg acceptor [5]. To address this challenge, Nakamura et al. introduced a post-growth process involving thermal annealing in the N_2_ atmosphere to break the Mg-H bond and diminish the concentration of H, successfully converting as-grown Mg-doped GaN into a p-type semiconductor [6]. Such a method has become the industrial standard post-growth process for p-type activation of Mg-doped GaN [7].

However, the progress of GaN-based devices is impeded by a traditionally produced Mg-doped GaN with a low hole concentration. This limitation stems from the high ionization energy of the Mg acceptor (~170 meV), and the activation efficiency of Mg-doped GaN at room temperature remains limited to approximately 1% [8]. Thus, increasing the Mg doping concentration has been a common strategy to achieve a high hole concentration in heavily Mg-doped GaN.

For heavily Mg-doped GaN, the passivation effect of H formed during the growth process also exists in the material. Thus, the post-growth process must be considered, and the redistribution of H during thermal annealing is worthy of attention [9].

In addition, the self-compensation effect within the heavily Mg-doped GaN is another factor that prevents it from achieving a high hole concentration. As the Mg concentration in GaN increases, exceeding 2~3 × 10^19^ cm^−3^, the internal hole concentration does not increase proportionally but rather decreases [10]. Theoretical calculations elucidated that nitrogen vacancies (V_N_) as donor-type defects play an important role in this self-compensation effect [11]. Therefore, the behavior of relevant compensation defects in heavily Mg-doped GaN during thermal annealing also needs to be investigated.

To find out the evolution of relevant defects (H and V_N_) in the heavily Mg-doped GaN during the post-growth process, we conducted experiments involving H ion implantation and post-implantation thermal annealing at different temperatures and in different atmospheres (N_2_ and NH_3_), which enabled us to delve into both factors that affect the heavily Mg-doped GaN. On the one hand, the redistribution characteristics of H ions in the material were analyzed through the results of secondary ion mass spectroscopy (SIMS). On the other hand, the behavior of H-related defects was investigated through SIMS results and their photoluminescence (PL) spectra. Additionally, the behavior of V_N_ was also analyzed by combining its PL spectra and surface properties which were revealed by its contact properties with metals and its ultraviolet photoelectron spectroscopy (UPS) and X-ray photoelectron spectroscopy (XPS) results. In particular, we compared the effect of two different thermal annealing atmospheres on the evolution of those relevant defects. By doing so, we aimed to provide crucial insights into the post-growth processes of heavily Mg-doped GaN.

## 2. Materials and Methods

Heavily Mg-doped GaN film was prepared through MOCVD. Initially, a 1.5 μm-thick unintentionally doped GaN layer was grown on a c-plane sapphire substrate via MOCVD. Subsequently, a 500 nm-thick heavily Mg-doped GaN layer was grown with a growth temperature of 950 °C, chamber pressure of 200 Torr, and duration of 60 min. Trimethylgallium (TMGa), NH_3_, and bis(cyclopentadienyl)magnesium (Cp_2_Mg) were respectively used as sources of Ga, N, and Mg, and the flow rate of them was respectively controlled at 13, 3000, 110 sccm, in which H_2_ was used as carrier gas. The Mg concentration of 1.1 × 10^20^ cm^−3^ in this heavily Mg-doped GaN film was detected by SIMS.

Subsequently, H ions were implanted vertically into the as-grown sample with an energy of 30 keV and a dose of 1 × 10^15^ cm^−2^, obtaining Sample H.

Followed by H ion implantation, thermal annealing was conducted in a MOCVD reactor. One set of four samples underwent post-implantation annealing in an N_2_ atmosphere, with the annealing temperatures respectively set at 600 °C for Sample HN6, 700 °C for Sample HN7, 800 °C for Sample HN8, and 900 °C for Sample HN9. Similarly, another set of four samples was annealed in an NH_3_ atmosphere, with temperatures controlled at 600 °C for Sample HA6, 700 °C for Sample HA7, 800 °C for Sample HA8, and 900 °C for Sample HA9. The annealing time was fixed at 20 min.

The depth profile of H concentration within each sample was characterized by SIMS utilizing the CAMECA ims-7f instrument (AMETEK, Berwyn, PA, USA).

PL was excited with the 325 nm line of an IK Series He-Cd laser (Kimmon Koha, Tokyo, Japan) at room temperature under continuous excitation (350 W/cm^2^) and acquired using a Spectro2500i spectrometer with a PIXIS:256E CCD detector (Princeton Instruments, Trenton, NJ, USA).

To characterize the contact properties between the semiconductor and metals (Ni/Au), the current–voltage (I–V) curves were carried by the circular transfer length method (CTLM). The CLTM pattern was designed with an inner radius of 110 μm and a gap distance of 10 μm. The electrode fabrication process involved e-beam deposition of Ni/Au (20 nm/20 nm) onto samples HN9 and HA9 after immersion in a hydrochloric acid solution (HCl: H_2_O = 1:1). The Ni/Au contacts were thermal annealed by rapid thermal annealing (RTA) in an oxygen atmosphere at temperature of 550 °C for 1 min.

Atomic force microscopy (AFM) utilizing Dimension 3100 (Veeco, Plainview, NY, USA) was employed for a surface morphology study. To elucidate the surface property of heavily Mg-doped GaN, UPS and XPS were conducted by the ESCALAB Xi+ photoelectron spectrometer (Thermo Fihser, Waltham, MA, USA). In XPS, the energy levels were referenced to the C 1s (284.5 eV) [12]. In UPS, an He I light source (21.22 eV) was used and the Fermi level of samples was calibrated by a clean-surfaced standard gold sample with a work function of 5.10 eV.

## 3. Results and Discussion

### 3.1. Redistribution of H Ions

**Figure 1:**
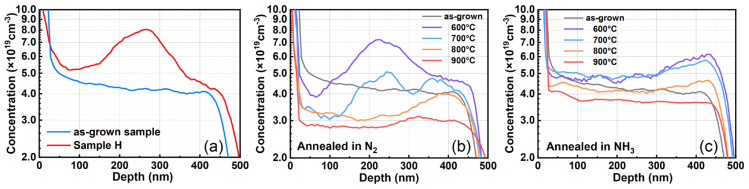
Secondary ion mass spectroscopy: depth profile of H concentration in (**a**) as-grown sample and Sample H without annealing after H ion implantation; (**b**) Sample HN6~HN9 annealed at 600~900 °C in N_2_ after H ion implantation; (**c**) Sample HA6~HA9 annealed at 600~900 °C in NH_3_ after H ion implantation.

Figure 1a illustrates the depth profiles of H concentration in the as-grown sample and Sample H (H-implanted sample). In the as-grown sample, the distribution of H is relatively uniform across depth. Conversely, in Sample H, a distinct peak is observed at a projection depth of 270 nm, with the highest concentration reaching 8 × 10^19^ cm^−3^ in the H concentration profile.

The SIMS result for Sample H reveals a notable trend that the H concentration exhibits higher levels within the depth range of 50~150 nm compared to a depth of 400~450 nm. This observation suggests a phenomenon characterized by heightened concentration near the surface and diminished concentration in the bulk. This result can be attributed to the light mass of H ions, leading to a significant backscattering effect during ion implantation. Consequently, the distribution of H in Sample H diverges from the standard Gaussian distribution, instead displaying a negatively skewed Pearson IV distribution [13,14]. Such a backscattering effect also leads to a lower maximum concentration of hydrogen in Sample H compared to the SRIM calculation result (as shown in Figure S1).

Figure 1b,c respectively depict the SIMS results of the H depth profile in samples subjected to post-implantation thermal annealing in N_2_ and NH_3_ atmospheres, respectively denoted as Sample HN6~NH9 and Sample HA6~HA9. A notable trend observed in these SIMS results is that, with increasing annealing temperature, the H ions implanted into samples gradually diffuse out of the material, regardless of whether the sample is thermal annealed in an N_2_ or NH_3_ atmosphere.

In both Sample HN9 and Sample HA9, the concentration of H exhibits a relatively uniform distribution across depth, consistent with the characteristics observed in the as-grown sample. This indicates that H ions acquire sufficient energy to escape from the heavily Mg-doped GaN at a temperature of 900 °C. Consequently, nearly all H ion implanted into the sample are observed to escape through thermal annealing at 900 °C, irrespective of the atmosphere (N_2_ or NH_3_).

Notably, when samples implanted with H ions undergo thermal annealing in N_2_, an H concentration peak is still observed around a depth of 250 nm within Sample HN6 and Sample HN7 annealed at temperatures of 600 °C and 700 °C. However, at temperatures of 800 °C and 900 °C, such a concentration peak is absent in Sample HN8 and Sample HN9. In contrast, when samples are thermal annealed in NH_3_, the concentration of H at the same depth diminishes rapidly, and no peak is observed at the low temperature of 600 °C.

This phenomenon indicates that diffusion of H ions is more effective when samples are thermal annealed in NH_3_ compared to N_2_. This also implies that there exist different processes of H diffusion from the material. Taking the thermal stability of N_2_ and NH_3_ into consideration, both the migration of H ions in the sample and the decomposition of NH_3_ in the chamber environment can occur simultaneously during thermal annealing at 600 °C [15]. Specifically, NH_3_ is more likely to decompose into certain molecules or ions with dangling bonds. Thus, the H ions near the surface have the opportunity to interact with the decomposed NH_3_ products, and this behavior accelerates their escape from the heavily Mg-doped GaN.

Furthermore, it can be clearly seen from the SIMS results that the H concentration in Sample HN6~HN9 and Sample HA6~HA7 displays a phenomenon characterized by diminished concentration near the surface and heightened concentration in the bulk. This redistribution is opposite to the trend observed in Sample H mentioned above. The reason behind this phenomenon may stem from the damage introduced to the surface and interior of GaN during the H ion implantation. These damages serve as channels through which H ions can more easily escape from the material.

However, in Sample HA8 and Sample HA9, the concentration of H near 50 nm is comparable to or even slightly higher than that at the depth of around 400 nm. This is because the majority (99%) of NH_3_ decomposes at temperatures ranging from 800 °C to 900 °C [16]. Consequently, a portion of the H produced by NH_3_ decomposition may either be adsorbed onto the GaN surface or penetrate into the material near the surface, resulting in a slightly higher concentration of H near the surface compared to the interior of Sample HA8 and Sample HA9. The reason behind this phenomenon is consistent with that observed in the H depth profiles in Sample HA6 and Sample HA9, where the H concentration peak disappears quickly.

Those results collectively indicate that the behavior of H in heavily Mg-doped GaN varies when thermal annealed in different atmospheres. NH_3_ can promote the diffusion of H in the heavily Mg-doped GaN during post-growth processes.

### 3.2. Behavior of Relevant Defects

**Figure 2:**
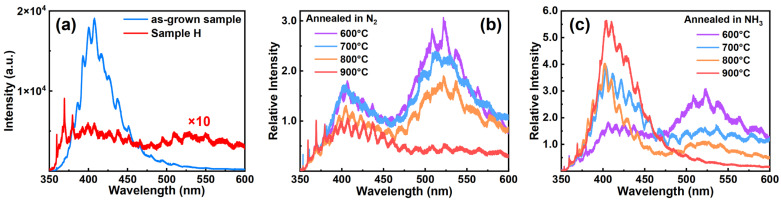
Photoluminescence spectra of (**a**) as-grown sample and Sample H without annealing after H ions irradiation; (**b**) Sample HN6~HN9 annealed at 600~900 °C in N_2_ after H ion implantation; (**c**) Sample HA6~HA9 annealed at 600~900 °C in NH_3_ after H ion implantation. Note: the PL intensity of Sample H in (**a**) is multiplied by a factor of 10 to clearly show its features, and the PL intensities of Sample HN6~HN9 and Sample HA6~HA9 in (**b**,**c**), respectively, are normalized to the maximum intensity of their corresponding NBE band.

Because of the thermal stability of N_2_ and decomposition of NH_3_, defects induced by ion implantation in those samples would undergo certain distinct processes during thermal annealing. The PL spectra of samples shown in Figure 2a–c can assist us in investigating the behavior of internal defects within the samples. The intensity oscillations in PL spectra result from the Fabry–Perot interference, which is commonly observed in film samples, because of the large difference in refractive indices among the sapphire substrate, GaN film, and air [17,18]. Combining the aforementioned discussion of SIMS results and certain surface analysis methods, we can find out the evolution of related defects through the changes observed in those PL spectra.

#### 3.2.1. H-Related Defects and Yellow Luminescence

Figure 2a compares the PL intensity of the as-grown sample and Sample H which has been subjected to H ion implantation but not yet thermally annealed. For the PL spectra of the as-grown sample, a dominant blue luminescence (BL) band is observed, centered at a wavelength of 410 nm (3.02 eV). This BL band, commonly observed in heavily Mg-doped GaN, is ascribed to the donor–acceptor pair (DAP) recombination involving an electron transition from an unknown deep donor to the shallow Mg_Ga_ acceptor [19]. However, the PL spectra of Sample H display three different luminescence bands: the near-band-edge (NBE) band, BL band, and yellow luminescence (YL) band. The peak wavelengths of these bands are respectively centered at 369 nm (3.36 eV), 410 nm (3.02 eV), and 525 nm (2.36 eV).

In comparison to the PL spectra of the as-grown sample, each luminescence band in the PL spectra of Sample H exhibits a notable reduction in intensity. The implantation of H ions into the material induces damage to both the surface and interior. Those damages do not only serve as channels for H out-diffusion as discussed above, but also function as non-radiative recombination centers. Consequently, the PL intensity of Sample H is dramatically diminished.

In particular, a new luminescence band is observed in the PL spectra of Sample H, that is, a broad YL band. It is worthy to be noted that lattice defects induced in GaN by ion implantation do not typically give rise to a YL band [20]. There is a persistent debate about the origin of the YL band in GaN, such as C, O, or H-related defects, Ga vacancies, and certain complexes of them [21,22,23,24,25]. However, the appearance of the YL band in Sample H which is subjected to H ion implantation strongly suggests its association with H-related defects within the heavily Mg-doped GaN.

Figure 2b,c, respectively, depict the PL spectra of Sample HN6~NH9 and Sample HA6~HA9. Given that those non-radiative centers induced by ion implantation would evolve when samples are thermal annealed in different conditions, their PL intensities are normalized to the maximum intensity of their corresponding NBE bands [26].

Regarding the YL band in PL spectra arising from H ion implantation, the relative intensity of the YL band among all samples shown in both Figure 2b,c gradually decreases with increasing annealing temperatures, regardless of whether thermal annealing in N_2_ or NH_3_. When the annealing temperature reaches 900 °C, the relative intensity of the YL band drops to a minimum in both Sample HN9 and Sample HA9.

Furthermore, such behavior of the YL band is nearly consistent with the redistribution characteristics of H ions in those samples, where H concentrations also decrease when increasing the annealing temperature. Therefore, we can correlate the YL band in PL spectra with H-related defects induced by H ion implantation in samples.

It should be noted that the decomposition of NH_3_ does not interfere with our conclusion. As discussed above, when the thermal annealing temperature is increased, the decomposition of NH_3_ is expected to occur. This results in higher hydrogen concentration near the surface compared to the interior of Sample HA8 and Sample HA9. However, Czernecki et al. have found that the PL spectra of Mg-doped GaN grown by MOCVD are not changed by annealing in an H_2_ + NH_3_ atmosphere [27]. Therefore, H ions generated from the decomposition of NH_3_ in our thermal annealing experiments actually do not impact the YL band. This might be due to the H ions from NH_3_ decomposition occupying different lattice locations in GaN compared to those introduced by ion implantation [28].

For the YL band in PL spectra, it appears after H ion implantation and disappears after the out-diffusion of H ions during thermal annealing. Thus, we conclude that the YL band is closely associated with H-related defects which would evolve in similar trends during thermal annealing in N_2_ and NH_3_.

#### 3.2.2. Nitrogen Vacancies and Blue Luminescence

Regarding BL bands in Figure 2b,c, their intensity exhibits contrasting trends under different post-implantation thermal annealing atmospheres. Specifically, the intensity of the BL band decreases in N_2_ but increases in NH_3_ with increasing annealing temperatures. When samples are annealed at 900 °C, the BL band intensity reaches a minimum in N_2_ but a maximum in NH_3_. Those distinct trends in the intensity of the BL band suggest that two opposing processes occurring within heavily Mg-doped GaN in N_2_ and NH_3_. Nakamura et al. attributed the decrease in BL band intensity in N_2_ to the decomposition of Mg-doped GaN [5]. Hence, there exists a close association between the surface property of the sample and the intensity of the BL band.

**Figure 3:**
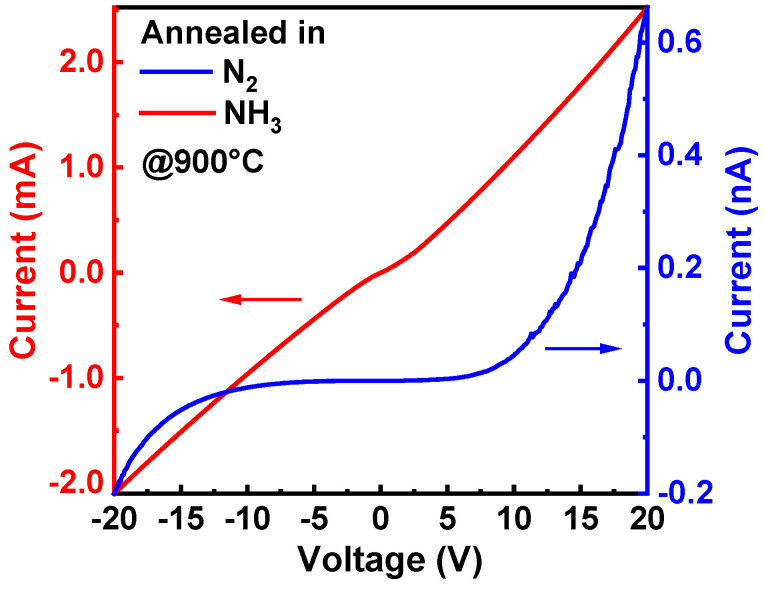
CTLM I–V measurement of Ni/Au contacts to Sample HN9 and HA9 with a gap distance of 10 μm.

The surface properties of semiconductors significantly affect their contact with metals. We e-beam evaporated Ni/Au (20 nm/20 nm) onto Sample HN9 and Sample HA9, followed by RTA in O_2_ for 1 min. The I–V curves in Figure 3, measured by CTLM with a gap distance of 10 μm, provide insights into the metal-semiconductor contact properties. Sample HN9, annealed in N_2_, forms a typical Schottky contact with metals; however, Sample HA9, annealed in NH_3_, forms an Ohmic contact with metals. This comparison directly shows that there are indeed certain differences between the surface properties of Sample HN9 and Sample HA9.

AFM measurements of Sample HN9 and Sample HA9 (shown in Figure S2) revealed that there are no distinct changes in surface morphology and roughness of Sample HN9 and Sample HA9. Because the size of H ions is too small, and dramatic degradation of surface morphology only occurs at the temperature around 1000 °C [29,30,31], thus, it is reasonable to expect that the impact of our post-implantation thermal annealing, conducted at 600 °C to 900 °C, on the surface morphology would be neglectable. Thus, the surface properties of Sample HN9 and Sample HA9 may differ on a smaller scale.

To further analyze the surface discrepancy between these two samples, we performed XPS and UPS measurements on Sample HN9 and Sample HA9 to explore the energy band and chemical bonds on their surface.

**Figure 4:**
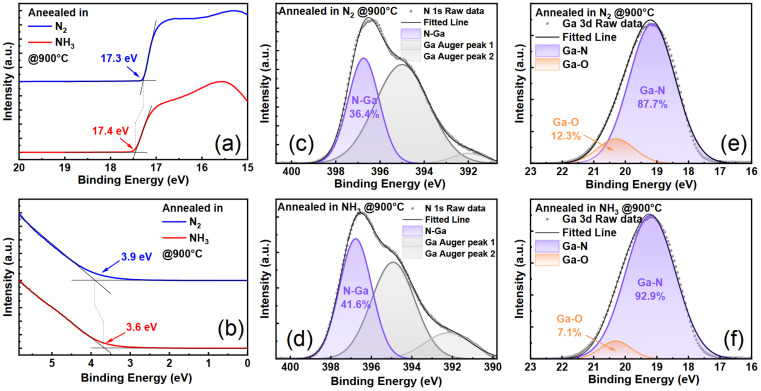
Ultraviolet photoelectron spectroscopy of HN9 sample and HA9 sample: secondary electron cutoff edge (**a**) and low binding energy band (**b**); X-ray photoelectron spectroscopy: N 1s energy level of HN9 sample (**c**) and HA9 sample (**d**); Ga 3d energy level of HN9 sample (**e**) and HA9 sample (**f**).

Figure 4a,b, respectively, depict the secondary electron cutoff (SECO) region and the region of low electron binding energy in UPS, in which the Fermi level corresponds to a binding energy of 0 eV. It is clearly observed that the SECO lies at 17.3 eV for Sample HN9 and 17.4 eV for Sample HA9, with a slight difference of 0.1 eV. Thus, it can be assumed that the vacuum level is the same for both Sample HN9 and Sample HA9 [32,33].

The part of low binding energy in UPS reveals the position of the valence band maximum (VBM) on the surface. The VBM positions of Sample HN9 and Sample HA9 were extrapolated to be situated at 3.9 eV and 3.6 eV below the Fermi level, respectively. Given the bandgap of 3.4 eV, the Fermi level lies within the conduction band minimum (CBM), indicating a significant downward bending of the energy band on the surface of both samples.

Actually, this phenomenon is commonly observed in Mg-doped GaN, and it is related to the presence of V_N_ on the surface which acts as donor-type defects in GaN [34]. Considering the low displacement energy of N atoms [35], a large amount of V_N_ may emerge on the surface during H ion implantation, resulting in a pronounced downward band bending of samples.

The data above show that the Fermi level of Sample HA9 is closer to the VBM compared to HN9; that is, the surface energy band bending of HA9 is slighter than that of HN9. Therefore, it can be inferred that the V_N_ in Sample HA9 is repaired during thermal annealing in NH_3_ because of its decomposition as mentioned above.

If there is fewer V_N_ on the surface of Sample HA9, corresponding conclusions should be drawn by delving into relevant information in the chemical bonds of the atoms on the surface. Thus, we perform the XPS measurement on Sample HN9 and Sample HA9.

The detailed XPS scan of the N 1s and Ga 3d peak is recorded to assess the bonding state of the surface atoms, as shown in Figure 4c–f. The N 1s peaks are deconvoluted into three contributions in all cases: an N-Ga ionization peak (397.0 ± 0.5 eV) and two Ga Auger peaks (395.0 ± 0.5 eV, 392.0 ± 0.5 eV) [36]. Similarly, the Ga 3d peaks are de-convoluted into two contributions in all cases: a Ga-N ionization peak (19.2 ± 0.5 eV) and a Ga-O ionization peak (20.3 ± 0.5 eV) [37].

The bonding ratio of atoms can be revealed by the integrated area ratio of each sub-peak. The proportion of N-Ga sub-peak in the N 1s energy level of Sample HA9 (41.5%) is higher than that of HN9 (36.4%), and, similarly, the proportion of Ga-N sub-peak in the Ga 3d energy level of Sample HA9 (93.9%) is higher than that of Sample HN9 (87.7%).

These findings jointly indicate a higher bonding ratio between Ga atoms and N atoms on the surface of Sample HA9 compared to Sample HN9, implying fewer V_N_ on the surface of Sample HA9 compared to Sample HN9. Furthermore, it is because of the reduction in V_N_ that the downward band bending effect is alleviated on the surface of Sample HA9. Consequently, it leads to its formation of Ohmic contact with metals rather than a Schottky contact as observed in HN9.

Combining the above surface analysis of samples and the contrasting trends of the BL band intensity in their PL spectra, it can be concluded that the decomposition of NH_3_ can supply N atoms to repair the V_N_ on the surface. By doing so, it can effectively prevent the decomposition of the sample surface at high temperatures. Thus, with increasing the thermal annealing temperature, the intensity of the BL band in PL spectra increases in NH_3_ but decreases in N_2_.

## 4. Conclusions

In this study, we revealed the evolution of H and V_N_ in the heavily Mg-doped GaN through H ion implantation followed by thermal annealing at different temperatures and in different atmospheres (N_2_ and NH_3_).

The characteristics of H ions redistribution vary when samples are annealed in different atmospheres. Due to the decomposition of NH_3_, thermal annealing in the NH_3_ atmosphere enhances the efficiency of H ions’ out-diffusion from the material compared to annealing in the N_2_ atmosphere.

We also found that H-related defects introduced by H ion implantation are directly related to the YL band in the PL spectra. This is because they always evolve in a similar trend during thermal annealing regardless of annealing in N_2_ or NH_3_. Specifically, both H concentration and YL band intensity tend to decrease with increasing annealing temperature.

By analyzing the evolution of the BL band and V_N_ which are primary compensation defects in the heavily Mg-doped GaN, we discovered that the decomposition of NH_3_ not only facilitates the faster escape of H ions from the material but also supplies N atoms to repair those V_N_ on the surface of heavily Mg-doped GaN.

Given that H and V_N_ are significant factors that respectively result in passivation and compensation effects in heavily Mg-doped GaN, with both effects hindering its further development, our findings could provide crucial insights for the post-growth process of heavily Mg-doped GaN.

## Data Availability

The data presented in this paper is available with the request from the corresponding author.

## Supplementary Materials

The following supporting information can be downloaded at: https://www.mdpi.com/article/10.3390/ma17112518/s1, Figure S1: The H depth profile in Sample H calculated by SRIM compared with that detected by SIMS; Figure S2: AFM images of Sample HN9 and Sample HA9.

## References

[1] Moustakas T.D., Paiella R. (2017). Optoelectronic Device Physics and Technology of Nitride Semiconductors from the UV to the Terahertz. Rep. Prog. Phys..

[2] (2014). The Nobel Prize in Physics. https://www.nobelprize.org/prizes/physics/2014/summary/.

[3] Kneissl M., Seong T.-Y., Han J., Amano H. (2019). The Emergence and Prospects of Deep-Ultraviolet Light-Emitting Diode Technologies. Nat. Photonics.

[4] Greco G., Iucolano F., Roccaforte F. (2018). Review of Technology for Normally-off HEMTs with p-GaN Gate. Mater. Sci. Semicond. Process..

[5] Nakamura S., Iwasa N., Senoh M., Mukai T. (1992). Hole Compensation Mechanism of P-Type GaN Films. Jpn. J. Appl. Phys..

[6] Nakamura S., Mukai T., Senoh M., Iwasa N. (1992). Thermal Annealing Effects on P-Type Mg-Doped GaN Films. Jpn. J. Appl. Phys..

[7] Nakamura S. (2015). Nobel Lecture: Background Story of the Invention of Efficient Blue InGaN Light Emitting Diodes. Rev. Mod. Phys..

[8] Nardo A., de Santi C., Carraro C., Sgarbossa F., Buffolo M., Diehle P., Gierth S., Altmann F., Hahn H., Fahle D. (2022). Laser-Induced Activation of Mg-Doped GaN: Quantitative Characterization and Analysis. J. Phys. D Appl. Phys..

[9] Sakurai H., Narita T., Omori M., Yamada S., Koura A., Iwinska M., Kataoka K., Horita M., Ikarashi N., Bockowski M. (2020). Redistribution of Mg and H Atoms in Mg-Implanted GaN through Ultra-High-Pressure Annealing. Appl. Phys. Express.

[10] Obloh H., Bachem K.H., Kaufmann U., Kunzer M., Maier M., Ramakrishnan A., Schlotter P. (1998). Self-Compensation in Mg Doped p-Type GaN Grown by MOCVD. J. Cryst. Growth.

[11] Lyons J.L., Janotti A., Van de Walle C.G. (2014). Effects of Hole Localization on Limiting *p* -Type Conductivity in Oxide and Nitride Semiconductors. J. Appl. Phys..

[12] Greczynski G., Hultman L. (2022). A Step-by-Step Guide to Perform X-ray Photoelectron Spectroscopy. J. Appl. Phys..

[13] Wilson R.G. (1980). The Pearson IV Distribution and Its Application to Ion Implanted Depth Profiles. Radiat. Eff..

[14] Lutsch A.G., Oliver D.N. (1983). Implantation through a Window with Medium to High Energy Ions. Microelectron. J..

[15] Shekhar R., Jensen K.F. (1997). Temperature Programmed Desorption Investigations of Hydrogen and Ammonia Reactions on GaN. Surf. Sci..

[16] Moon Y.-T., Kim D.-J., Park J.-S., Oh J.-T., Lee J.-M., Park S.-J. (2004). Recovery of Dry-Etch-Induced Surface Damage on Mg-Doped GaN by NH_3_ Ambient Thermal Annealing. J. Vac. Sci. Technol. B Microelectron. Nanometer Struct. Process. Meas. Phenom..

[17] Billeb A., Grieshaber W., Stocker D., Schubert E.F., Karlicek R.F. (1997). Microcavity Effects in GaN Epitaxial Films and in Ag/GaN/Sapphire Structures. Appl. Phys. Lett..

[18] Hums C., Finger T., Hempel T., Christen J., Dadgar A., Hoffmann A., Krost A. (2007). Fabry-Perot Effects in InGaN/GaN Heterostructures on Si-Substrate. J. Appl. Phys..

[19] Nayak S., Gupta M., Waghmare U.V., Shivaprasad S.M. (2019). Origin of Blue Luminescence in Mg-Doped Ga N. Phys. Rev. Appl..

[20] Kucheyev S.O., Toth M., Phillips M.R., Williams J.S., Jagadish C., Li G. (2002). Chemical Origin of the Yellow Luminescence in GaN. J. Appl. Phys..

[21] Lyons J.L., Janotti A., Van De Walle C.G. (2010). Carbon Impurities and the Yellow Luminescence in GaN. Appl. Phys. Lett..

[22] Soh C.B., Chua S.J., Lim H.F., Chi D.Z., Tripathy S., Liu W. (2004). Assignment of Deep Levels Causing Yellow Luminescence in GaN. J. Appl. Phys..

[23] Yan Q., Janotti A., Scheffler M., Van De Walle C.G. (2012). Role of Nitrogen Vacancies in the Luminescence of Mg-Doped GaN. Appl. Phys. Lett..

[24] Reshchikov M.A. (2023). On the Origin of the Yellow Luminescence Band in GaN. Phys. Status Solidi (B).

[25] Zhang R., Kuech T.F. (1998). Hydrogen Induced Yellow Luminescence in GaN Grown by Halide Vapor Phase Epitaxy. J. Electron. Mater..

[26] Reshchikov M.A., Morkoç H. (2005). Luminescence Properties of Defects in GaN. J. Appl. Phys..

[27] Czernecki R., Grzanka E., Jakiela R., Grzanka S., Skierbiszewski C., Turski H., Perlin P., Suski T., Donimirski K., Leszczynski M. (2018). Hydrogen Diffusion in GaN:Mg and GaN:Si. J. Alloys Compd..

[28] Wampler W.R., Myers S.M., Wright A.F., Barbour J.C., Seager C.H., Han J. (2001). Lattice Location of Hydrogen in Mg Doped GaN. J. Appl. Phys..

[29] Choi H.W., Rana M.A., Chua S.J., Osipowicz T., Pan J.S. (2002). Surface Analysis of GaN Decomposition. Semicond. Sci. Technol..

[30] Bchetnia A., Kemis I., Touré A., Fathallah W., Boufaden T., Jani B.E. (2008). GaN Thermal Decomposition in N_2_ AP-MOCVD Environment. Semicond. Sci. Technol..

[31] Koleske D.D., Wickenden A.E., Henry R.L., Culbertson J.C., Twigg M.E. (2001). GaN Decomposition in H_2_ and N_2_ at MOVPE Temperatures and Pressures. J. Cryst. Growth.

[32] Grodzicki M., Mazur P., Ciszewski A. (2018). Changes of Electronic Properties of P-GaN(0 0 0 1) Surface after Low-Energy N+-Ion Bombardment. Appl. Surf. Sci..

[33] Mahat M.R., Talik N.A., Abd Rahman M.N., Anuar M.A., Allif K., Azman A., Nakajima H., Shuhaimi A., Abd Majid W.H. (2020). Electronic Surface, Optical and Electrical Properties of p-GaN Activated via in-Situ MOCVD and Ex-Situ Thermal Annealing in InGaN/GaN LED. Mater. Sci. Semicond. Process..

[34] Lin Y.-J., Chu Y.-L. (2005). Effect of Reactive Ion Etching-Induced Defects on the Surface Band Bending of Heavily Mg-Doped p-Type GaN. J. Appl. Phys..

[35] Usman M., Hallén A., Nazir A. (2015). Ion Implantation Induced Nitrogen Defects in GaN. J. Phys. D Appl. Phys..

[36] Moldovan G., Roe M.J., Harrison I., Kappers M., Humphreys C.J., Brown P.D. (2006). Effects of KOH Etching on the Properties of Ga-Polar n-GaN Surfaces. Philos. Mag..

[37] Jiao W., Kong W., Li J., Collar K., Kim T.-H., Losurdo M., Brown A.S. (2016). Characterization of MBE-Grown InAlN/GaN Heterostructure Valence Band Offsets with Varying in Composition. AIP Adv..

