# Supplementary material for: Research on Evolution of Relevant Defects in Heavily Mg-Doped GaN by H Ion Implantation Followed by Thermal Annealing
Source: Materials (Basel). 2024 May 23;17(11):2518. doi: 10.3390/ma17112518 (PMC11173091; doi:10.3390/ma17112518)
Supplement: Supplementary file 1 [file materials-17-02518-s001.zip › materials-2999125-supplementary.pdf]

Supplementary Materials for Research on evolution of relevant defects in heavily Mg-doped GaN by H ions implantation followed by thermal annealing

Figure S1 illustrates the depth profile of H implanted into Mg doped-GaN calculated by SRIM and detected by SIMS. The calculating mode of Detailed Calculation with Full Damage Cascades at an energy of 30 keV was used in SRIM calculation. And considering the experimental dose of 1×10^{15} cm^{-2}, we calculated the H depth profile by multiplying the SRIM-calculated ion range by the experimental dose. The implantation energy and dose used in the simulation matched those used in our experiments.

SRIM result shows a projection depth (R_p) of 225 nm and a maximum hydrogen concentration of 11.9×10^{19} cm^{-3}. However, according to SIMS result, the projection depth of H in Sample H is 270 nm and the maximum hydrogen concentration is 8×10^{19} cm^{-3}.

For projection depth, experimental value is larger than calculated value. Because of the small volume of hydrogen and the vertical implantation angle, the channel effect would happen in the process of H ions implantation, which would lead to larger R_p in experiment.

For maximum hydrogen concentration, experimental value is lower than calculated value. Because of the light mass of hydrogen, backscattering effect would be prominent near the surface of material.

This would result in a higher concentration near the surface and a lower concentration in deeper layer, as we mentioned and discussed in the text.

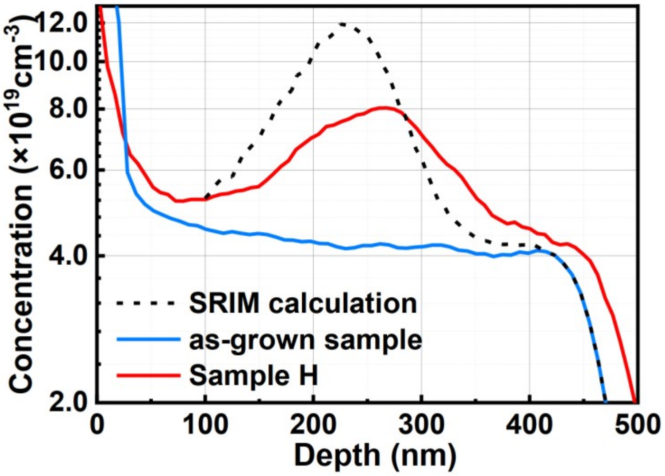

Figure S1 Depth profile of H concentration in as-grown sample and Sample H detected by SIMS. The H depth profile of SRIM calculation result is shown as black dashed line.

The AFM images of Sample HN9 and Sample HA9 are shown in Figure S2. The root-mean square (RMS) roughness values are acquired in an area of 2 μm×2 μm, which are 0.505 nm for Sample HN9 and 0.401 nm for Sample HA9.

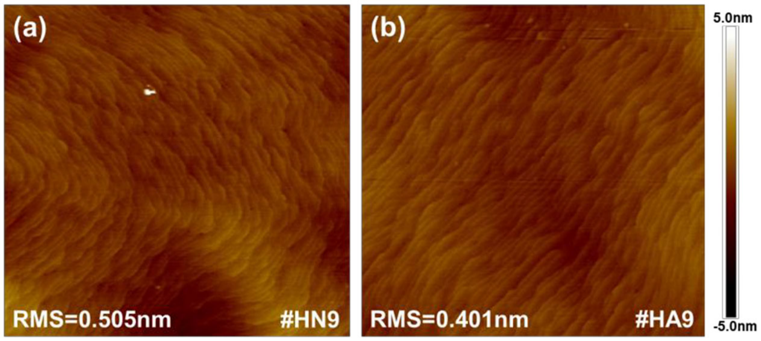

Figure S2 AFM images of (a) Sample HN9 and (b) Sample HA9.
